# Identification of a three miRNA signature as a novel potential prognostic biomarker in patients with bladder cancer

**DOI:** 10.18632/oncotarget.22318

**Published:** 2017-11-06

**Authors:** Fei Peng, Hui Li, Hailang Xiao, Ling Li, Yan Li, Yi Wu

**Affiliations:** ^1^ Department of Laboratory, The First Affiliated Hospital of Hunan Normal University, Hunan Provincial People's Hospital, Changsha, China; ^2^ Reproductive Department, Xiangya Hospital, Central South University, Changsha, China; ^3^ Nephrology Department, The First Affiliated Hospital of Hunan Normal University, Hunan Provincial People's Hospital, Changsha, China; ^4^ Department of Paediatrics, The First Affiliated Hospital of Hunan Normal University, Hunan Provincial People's Hospital, Changsha, China

**Keywords:** BLCA, miRNA, prognosis, biomarker, TCGA

## Abstract

There is not a good biomaker that is closely related to survival time for bladder cancer(BLCA), The aim of the study is to identify a miRNA signature that could predict prognosis in BLCA patients according to the data from The Cancer Genome Atlas (TCGA). a total of 377 BLCA patients were finally enrolled in the study. The three miRNA signature was identified by Multivariate Cox proportional hazards analyses that common clinical variables were controled. The three microRNA signature showed greater predicting prognosis capacity for predicting 5-year survival in BLCA with an AUC of 0.664, 0.681 and 0.668 in Train set, Test set and Total set respectively. Furthermore, there was a significant difference between high score and low score in Total set(P=3e-05), Test set(P=0.00435) and Train set(P=0.00143), respectively. Therefore, these results provided a new prospect for prognostic biomarker of BLCA.

## INTRODUCTION

Bladder cancer is the ninth most common malignancies worldwide [1]. 76, 960 new cases (4.6% of all new cancer cases) and 16, 390 deaths of bladder cancer were estimated in the United States during 2016 [2]. It brings a heavy economic burden to our society. In the USA, the annual national cost of bladder cancer care has outpaced inflation in recent years and is expected to reach $US5.25 billion ($US4.91 billion, 2010 values) in 2020 [3]. Bladder cancer has different biological characteristics. Superficial tumors are presented in 70% newly diagnosed patients which are usually not life threatening but tend to recur. Other 30% patients present with muscle-invasive status have a high risk of death from distant metastases. The invasive cystoscopy is the most common methods to detect suspicious bladder cancer because of the low sensitivity of other diagnostic tests like urine cytology [4]. Specific and sensitive biomarkers that can provide reliable information for tumor diagnosis or the prognosis may allow a more precise assessment and achieve a better-targeted effective therapy for bladder cancer. It is also of great importance to help illuminate the mechanism of bladder cancer development and progression.

MicroRNAs (miRNAs) are a family of short (typically 18–25 nucleotides), single-stranded and highly conserved non-coding RNAs that play an important role in the regulation of expression and function of eukaryotic genomes. They can bind to the 3′-untranslated region of target mRNAs through base pairing, resulting in mRNA degradation or translational repression [5]. Studies showed that miRNAs play a critical role in a variety of biological processes, such as cellular proliferation, apoptosis, migration, and angiogenesis [6–9]. All these processes are strongly related to tumor formation and development, which indicate a significant role of miRNAs in the pathogenesis of cancers. miR-17-92 is found overexpressed in human lung cancers and enhanced cell proliferation [10]. Five miRNAs (miR-185, miR-20a, miR-210, miR-25 and miR-92b) were up-regulated in gastric cancer and might serve as a diagnostic marker [11]. Other studies also revealed the relationship between miRNAs and other kinds of cancers, such as breast cancer [12], colorectal cancer [13]and pancreatic cancer [14]. Several studies have demonstrated the value of miRNAs as a prognostic marker in bladder cancer [15, 16]. However, most of the studies were carried out based on small number of patients or focused on just a specific miRNA. Here, we aimed to conduct the research using the dataset retrieved from The Cancer Genome Atlas (TCGA, http://cancergenome.nih.gov/) to identify a panel of miRNA signature which could predict prognosis in bladder cancer.

## RESULTS

### Characters of the datasets

A total of 437 miRNA expression profiles (level 3 data) were obtained from TCGA. In miRNA profiles, there are 1881 human miRNAs in BLCA samples(n=418) and normal tissues(n=19). The clinical data for those patients obtained from TCGA were available. According to included criteria, a total of 377 BLCA patients were finally enrolled in the study. For subsequent analysis, we randomly divided the total patients into the training set (n=189) and testing set (n=188) respectively. There was no significant difference on the clinical covariates between the two sets (P > 0.05). (Table 1).

**Table 1:** Characteristics of training set and testing set

covariates		Total	Training set	Testing set	P-value
		n=377	n=189	n=188	
age	≤65	152	81	71	0.345
	>65	225	108	117	
Pathologic stage	I-II	118	60	58	0.671
	III	130	67	63	
	IV	129	62	67	
Pathology T stage	T0-T2	108	57	51	0.675
	T3	182	91	91	
	T4	56	28	28	
	NA	30	12	18	
Pathology N stage	N0	215	114	101	0.584
	N1	43	19	24	
	N2	74	38	36	
	N3	7	2	5	
	NX	34	14	20	
	NA	4	2	2	
Pathology M stage	M0	173	92	81	0.598
	M1	9	3	6	
	MX	192	93	99	
	NA	2	1	1	
Gender	Male	279	136	143	0.363
	Female	98	53	45	
Radiation therapy	NO	338	166	172	0.502
	YES	19	11	8	
	NA	20	12	8	
Race	Asian	39	24	15	
	White	315	153	162	0.305
	Black or african american	23	12	11	
Status	Dead	167	81	86	0.573
	Survive	210	108	102	

### Identification of differentially expressed miRNAs in BLCA patients

Analysis of miRNA expression profiles in BLCA patient tissues (n = 418) compared with normal tissues (n = 19) identified a total of 392 differentially expressed miRNAs (logFC > 1 or logFC < −1, P < 0.05 after FDR adjustment). Of these, 295 miRNAs were overexpressed in BLCA patients(Supplementary Table 1), and 97 miRNAs were downexpressed in BLCA patients. (Supplementary Table 2).

### The miRNA signature risk score as an independent indicator for BLCA prognosis

**Figure 1:**
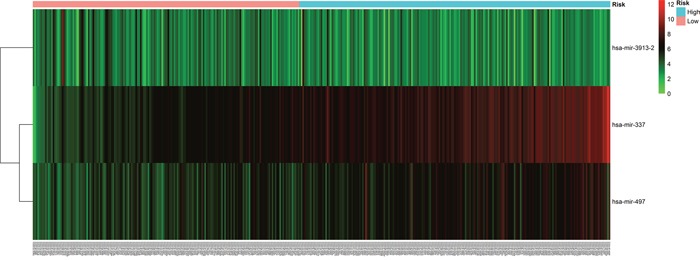
Three miRNA expressed in the score of BLCA patients

The risk score for each patient was calculated based on the 3 miRNAs. we developed a three microRNA signature. The miRNAs expression level was as the log2 reads per million of total aligned miRNA reads. The prognostic score was calculated as follows: Prognostic-score = (1.75×expression level of hsa-mir-337) + (−0.75×expression level of hsa-mir-3913-2)+(0.86 ×expression level of hsa-mir-497), 377 PC patients were classified into a high score group (n = 189) and low score group (n = 188). The risky miRNAs(hsa-mir-337 and hsa-mir-497) exhibit high expression in high score group, while hsa-mir-3913-2 didn't have obvious significance in both high and low score group(Figure 1). And the patients in the high score group suffered significantly worse survival time than those in low score group (Figure 1).

**Figure 2:**
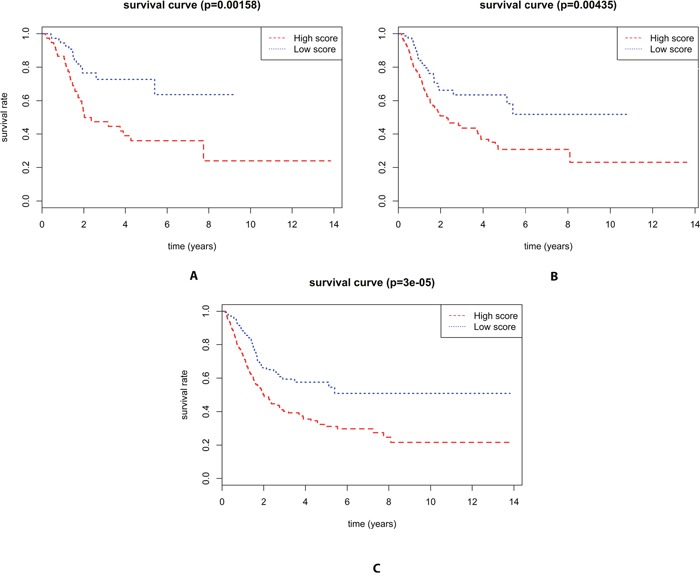
Kaplan–Meier curve for three miRNA signature and the survival time of BLCA patients **(A)** There is a significant difference between high score and low score in the survival time of BLCA patients in the Training set(*P*=0.00143) **(B)** There is a significant difference between high score and low score in the survival time of BLCA patients in the Testing set(*P*=0.00435) **(C)** There is a significant difference between high score and low score in the survival time of BLCA patients in the Total set(*P*=3e-05).

The univariate Cox regression analyses showed that Pathologic stage(P= 7.137E-07), Pathology T stage(P=0.0002), Pathology N stage(P=2.6971E-07) and the three microRNA signature((P=9.815E-07) were significantly related with survival time of BLCA patients, and the multivariate Cox regression analyses revealed that miRNA model scores was an independent prognostic factor(Table 2). In addition, the cut-off value was 14.05851 according to Train set and there was a significant difference between high score and low score in Total set(P=3e-05), Test set(P=0.00435) and Train set(P=0.00143), respectively(Figure 2). Morever, we found high score in the three microRNA signature had a shorten survival time in all groups(Supplementary Figure 3-11) except Female group(P=0.194)(Supplementary Figure 1) and Radiation therapy groupand(P=0.317) (Supplementary Figure 2). However, there was a significant difference in the remaining groups(Supplementary Figure 3-11)

**Table 2:** The association of clinical factors and the miRNA signature score with survival time in BLCA patients

variables	Univariate analysis		Multivariate analysis	
	HR(95%CI)	P value	HR(95%CI)	P value
Age(>65 vs ≤65)	2.270	0.0116		
Pathologic stage	1.821	7.137E-07	1.197	0.4263
Pathology T stage	1.621	0.0002	1.25	0.1738
Pathology N stage	1.585	2.6971E-07	1.285	0.1070
Gender(Male vs Female)	0.819	0.2986		
Radiation therapy(Yes vs No)	0.951	0.9123		
race(white vs Non-white)	1.221	0.6547		
miRNA model scores(High score vs Low score	7.101	9.815E-07	5.972	1.50E-05

**Figure 3:**
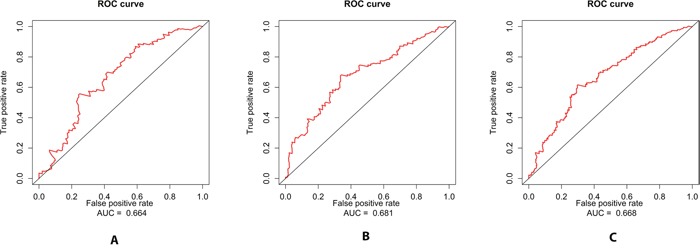
ROC curves for the three miRNA signature in predicting 5-year survival rate in BLCA patients **(A)** The ROC curve for predicting 5-year survival had an AUC of 0.664 in the Training set. **(B)** The ROC curve for predicting 5-year survival had an AUC of 0.681 in the Testing set. **(C)** The ROC curve for predicting 5-year survival had an AUC of 0.668 in the Total set.

Furthermore, The three microRNA signature showed greater predicting prognosis capacity for predicting 5-year survival in BLCA with an AUC of 0.664, 0.681 and 0.668 in Train set, Test set and Total set respectively(Figure 3). We also evaluated the three microRNA signature on the each subgroup of clinical characteristics and found it was significantly predictive for predicting 5-year survival in all groups (Supplementary Figure 12-22).

### Target prediction and functional enrichment of the three microRNA signature in BLCA

**Figure 4:**
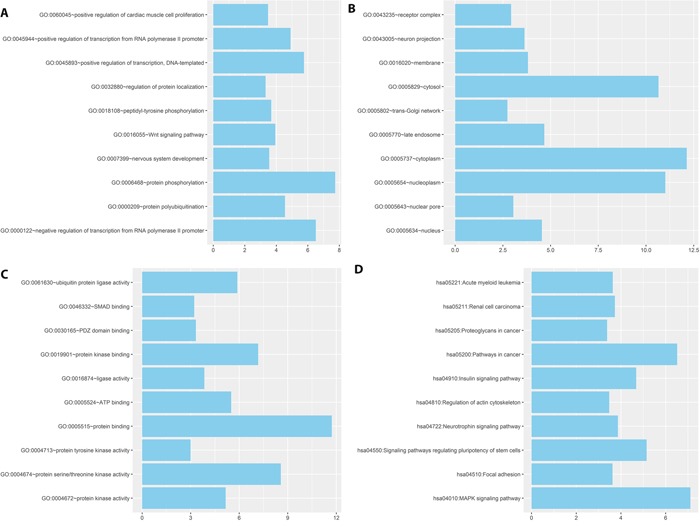
The top ten of GO term and pathway by target genes of three miRNA signature in BLCA patients **(A)** GO BP categories of three miRNA signature. **(B)** GO CC categories of three miRNA signature. **(C)** GO MF categories of three miRNA signature. **(D)** KEGG pathway of three miRNA signature.

The numbers of the target genes of the three miRNAs were 1034 and 1892, which were predicted by two data sets using miRanda and Targetscan. A total of 853 target genes were included in the two data sets (Supplementary Tables 3). We performed enrichment analyses to elucidate the biological function of target genes of the three microRNA signature. Finally, Gene ontology (GO) analysis revealed that there were 35 of the proteins were associated with biological process (BP)(Supplementary Tables 4, 46 of the proteins with cellular component(CC) (Supplementary Tables 4), and 56 of the proteins with molecular function (MF)(Supplementary Tables 4), respectively. The top ten enriched functional analysis was shown in Figure 4. The top of enriched biological process, cellular component and molecular function were protein phosphorylation, cytoplasm and protein binding respectively. Therefore, a total of 48 KEGG pathways were enriched by the three microRNA signature(Supplementary Tables 5). The top enriched KEGG pathway was the MAPK signaling pathway (Figure 4).

## DISCUSSION

MicroRNAs (miRNAs) are small non-coding RNAs that regulate gene expression by binding to the 3′-untranslated region of their target mRNAs [17]. Morever, miRNAs have been recognized as important intervention targets and predictive tools for various human cancers because of the stability and convenience of miRNA detection [18, 19]. Xu et al [20]found miR-33b-3p can improve the efficacy of chemotherapies for treating lung cancers, Cecene et al [21]indicated that miR-195 can be a clinically useful biomarker in Turkish breast cancer patients. However, there is not a excellent indicator to predict the survival time for BLCA patients. Hence, we made a three miRNA signature as to evaluate the prognosis of BLCA patients.

In this study, We applyed Multivariate Cox proportional hazards analyses that after controlling for common clinical variables to obtain the miRNA signature which can predict the prognosis of BLCA patients. After screening the best miRNA signature by the methor of AIC, A three miRNA signature consisting of hsa-mir-337, hsa-mir-3913-2 and hsa-mir-497 was identified as an independent predictor for prognosis of BLCA. There was a significant difference between high score and low score in all groups except the groups of radiation therapy, non-white and female. Conclusion should be cautious to confirm in the radiation therapy group due to only 19 patients are obtained in this group. Due to different clinical treatments could affect the relapse-free survival (RFS) and other survival endpoints of the patients. the conclusion that treated with Radiation therapy doesn't make much sense. In addition, the score of the three miRNA signature was not related to the survival time of female group and non-white group in BLCA patients according to our analysis. As for 5-year survival rate, the three miRNA signature have a good performance in all groups. The AUC of ROC curve for the three miRNA signature predicting 5-year survival rate were 0.664, 0.681 and 0.668 in the Training set, Testing set and Total set, respectively. However, the AUC value of ROC carve was not large that indicated the three miRNA signature for predicting 5-year survival needs to improve the accuracy and the specificity. Furthermore, we found only the score of three miRNA signature had a close association with the survival time of BLCA patients through the multivariate Cox regression analysis. For other clinical characteristics, no significant difference was found in the multivariate Cox regression analysis. That is to say, the three miRNA signature can be regarded as the only independent predictor for the patients of BLCA which means our miRNA signature is closely related to the survival time of BLCA. Wang et al [22]indicated that hsa-miR-337-3p expression did not dramatically affect gastric cancer cell proliferation, but transfection of the hsa-miR-337-3p mimic did reduce gastric cancer cell invasion capacity, Jiang et al [23]found that MiR-497 directly targeted 3′-UTR of Nrdp1 mRNA to inhibit its translation.

A total of 853 target genes were included in the two database of miRanda and Targetscan. Enrichment and function analysis was made, because we want to figure out the progression how a normal cell developed into a cancer cell. In enrichment and function analysis of DAVID database, our results shows that the top of enriched biological process, cellular component and molecular function were protein phosphorylation, cytoplasm and protein binding, respectively. As for protein phosphorylation, many studies have indicated it was closely related to cancer progress [24–25]. For KEGG pathway analysis, the most important signaling pathways was MAPK. It participate in the process of cancer development, such as endometrial cancer [26], lung cancer [27]. In addition, Kakkara et al [28]showed that MAPK pathways play a important role for altered signalling in DNT pathogenesis and targeted therapies. Liu et al [29] found that B7-H1 regulates p38 MAPK activation via association with DNA-PKcs.

Although the three miRNA signature is a excellent predictor for survival time of BLCA patients, some limitation is also existed. Firstly, there are only 377 patients of BLCA included in our study from TCGA that needs a validation or more cases to confirm. Secondly, the AUC of ROC curve in all groups is between 0.5 and 0.7 that means the three miRNA signature didin't perform very well in predicting 5-year survival rate in BLCA patients. In addition, the three miRNA signature was suitable for most subgroups of BLCA patients. However, there was no association between the three miRNA signature and two subgroups of female group and non-white group.

## MATERIALS AND METHODS

### Data sources and screening

The miRNA expression profiles (level 3 data) and corresponding clinical data for BLCA patients were obtained from TCGA data portal. Both the miRNA profiles data and clinical data of BLCA are publically available and open-access. These patients’ extended demographics were characterized by the TCGA. The patients were included in the study to meet the following criteria: (1) patients with fully characterized (clinical data and miRNA profiles) tumors; (2) patients with at least 1 month of survival time.

### Identification of differentially expressed miRNAs between BLCA and normal tissue

To identify miRNAs differentially expressed between BLCA and normal tissues, the raw counts of miRNA expression obtained from the TCGA dataset that consist of 418 BLCA samples and 19 normal tissue. One miRNA expression filter was miRNAs expressed in at least two normal or tumor samples. The expression differences were characterized by logFC(|log 2 fold change|>1) and associated P-values(P<0.05). The analysis was performed using the R package of edgeR [30]. Furthermore, the R package of pheatmap was used to draw the Heatmap.

### Survival analysis and ROC curve of prognostic model

First of all, the expression level of each miRNA was log2 transformed for further analysis. 377 BLCA patients were random divided into Training set and Testing set. Univariate Cox proportional hazards regression with significance level set as 0.001 was performed to find out the miRNAs significantly associated with survival time in the Training set. Multivariate Cox proportional hazards analyses were also conducted to evaluate the independent prognostic value of the miRNA signature after controlling for common clinical variables in the Training set. Akaike information criterion [31](AIC) was used to choose the best miRNA signature which was not only associated with survival time in Training set but also in Testing set. we used the median value of miRNA signature in Training set as cut off value and divided Training set, Testing set and Total set into high score group and low score group, respectively. Kaplan-Meier curves were used to estimate the survival for BLCA patients with high score or low score and ROC curve was adopt to predict the 5-year survival rate for BLCA patients with high score or low score. Chi-square test was used to evaluate the difference between Training set and Testing set and P value was set as 0.05. All analysis were performed using R (Packages: survival and survival ROC).

### Target prediction and enrichment analysis

The target genes of miRNAs were predicted by two programs including miRanda and Targetscan. The target genes were selected by miRanda and Targetscan that the final target genes were selected were included in all the two data sets). The enrichment analysis of these target genes was analyzed using DAVID online analysis [32] (https://david.ncifcrf.gov/). The gene sets containing less than 5 genes overlapping were removed from the DAVID analysis, and analysis for significance was determined when P values(P<0.05).

## SUPPLEMENTARY MATERIALS FIGURES AND TABLES












