# Supplementary material for: Identification of a three miRNA signature as a novel potential prognostic biomarker in patients with bladder cancer
Source: Oncotarget. 2017 Nov 6;8(62):105553–60. doi: 10.18632/oncotarget.22318 (PMC5739658; doi:10.18632/oncotarget.22318)
Supplement: Supplementary file 1 [file oncotarget-08-105553-s001.pdf]

Identification of a three miRNA signature as a novel potential prognostic biomarker in patients with blaader cancer

SUPPLEMENTARY MATERIALS

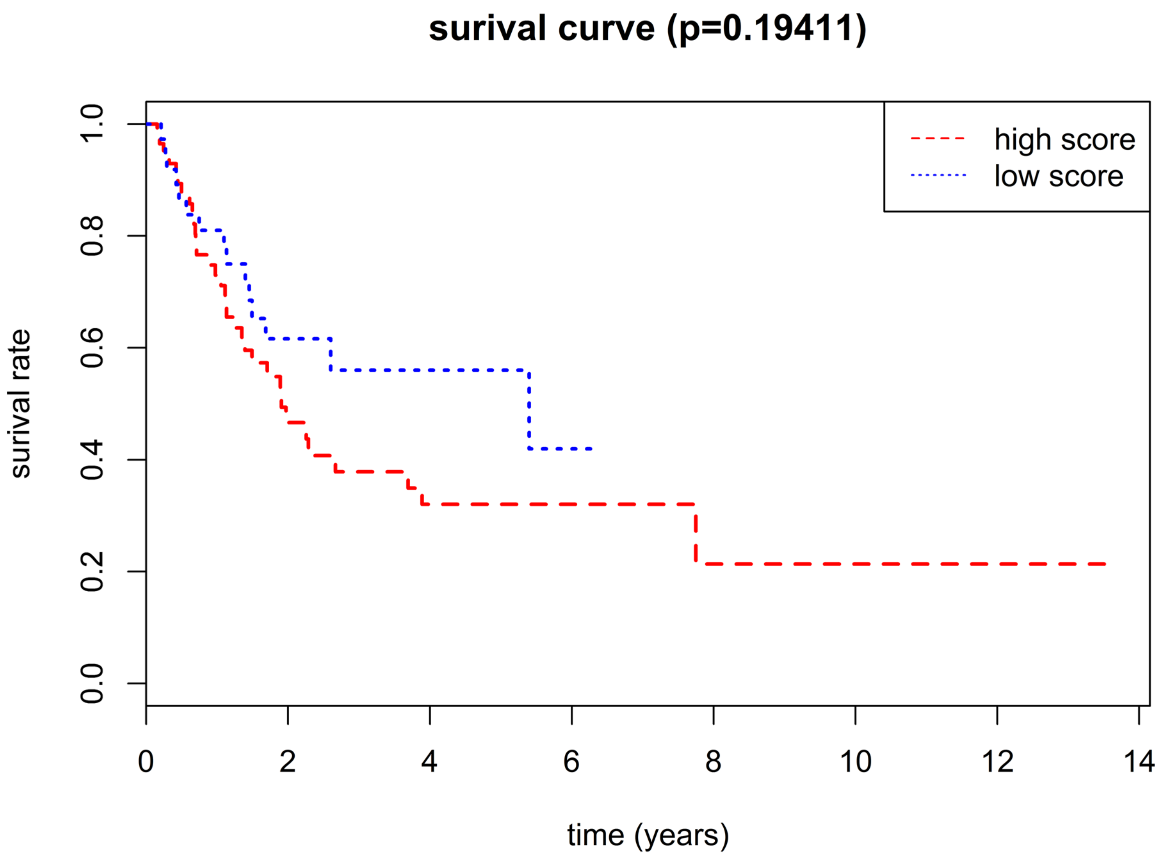

Supplementary Figure 1: Kaplan–Meier curve for the three-miRNA signature and the survival time of patients with BLCA in female group.

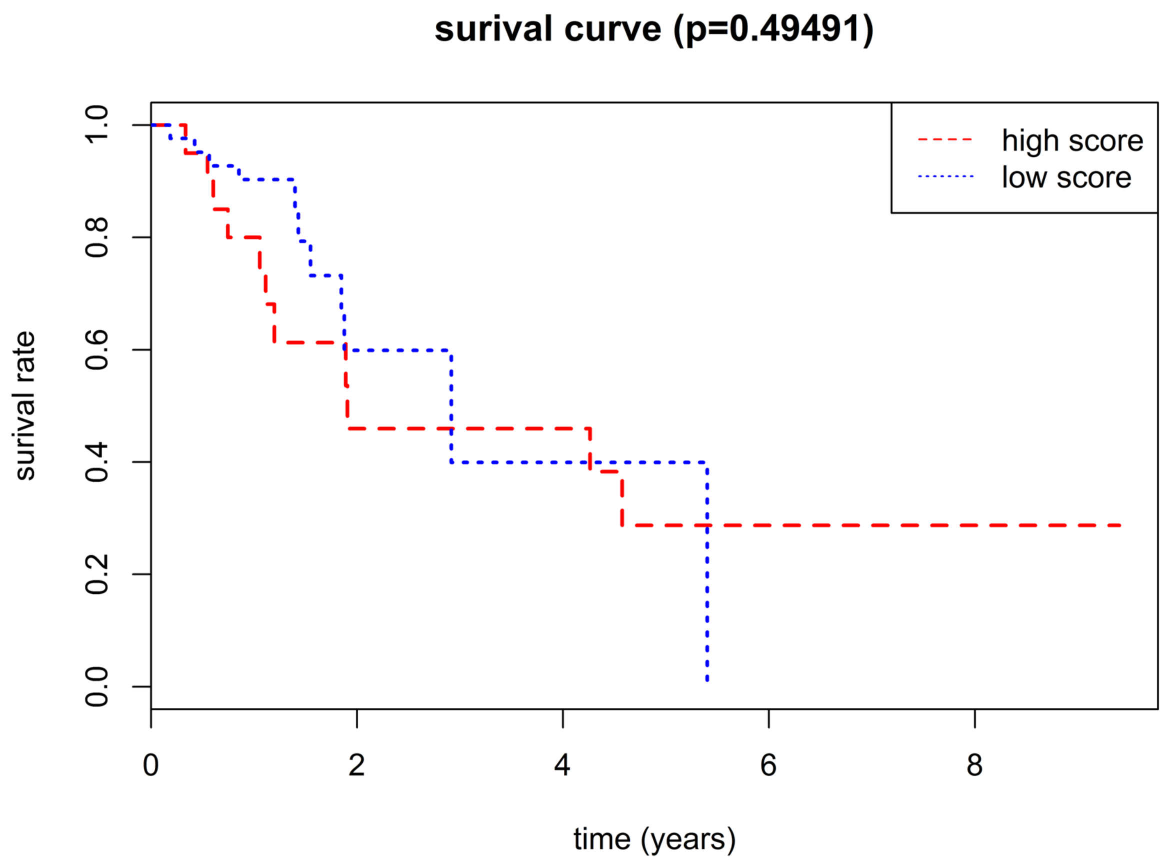

Supplementary Figure 2: Kaplan–Meier curve for the three-miRNA signature and the survival time of patients with BLCA in non-Caucasians group.

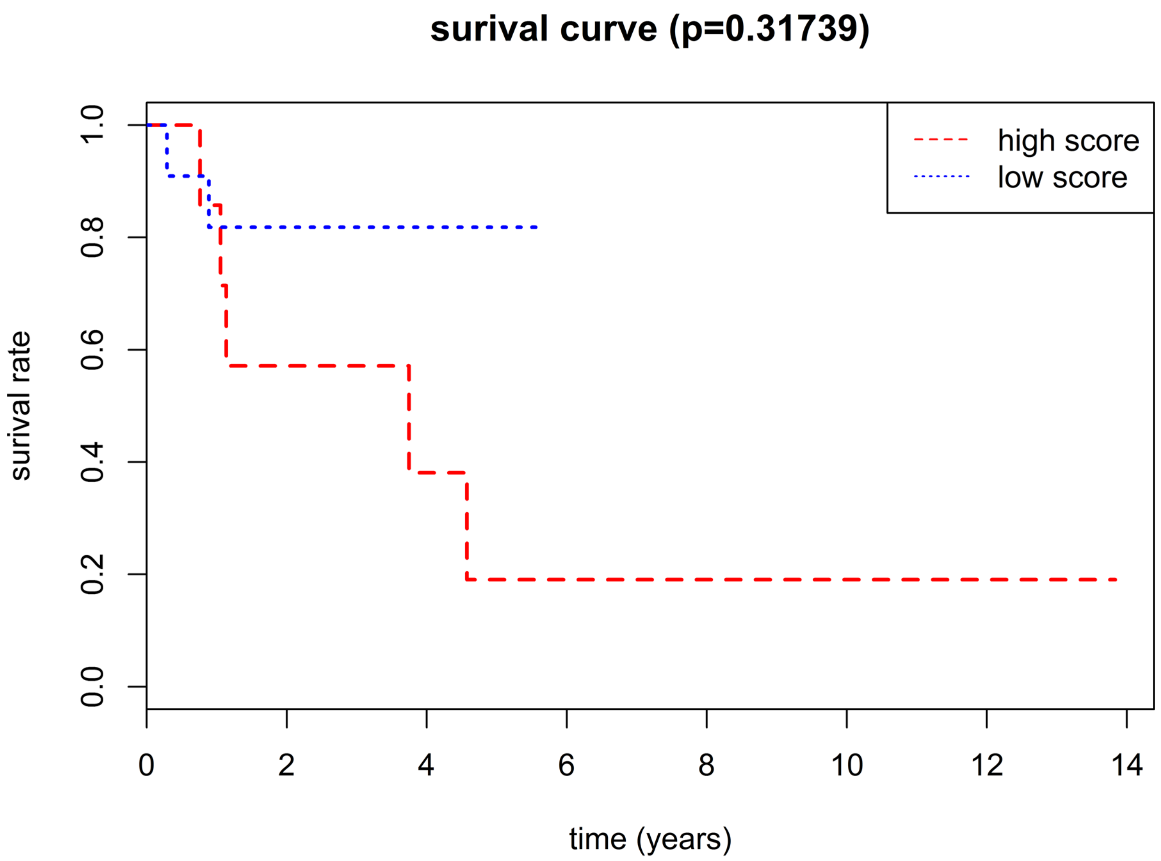

Supplementary Figure 3: Kaplan–Meier curve for the three-miRNA signature and the survival time of patients with BLCA in the radiation therapy group.

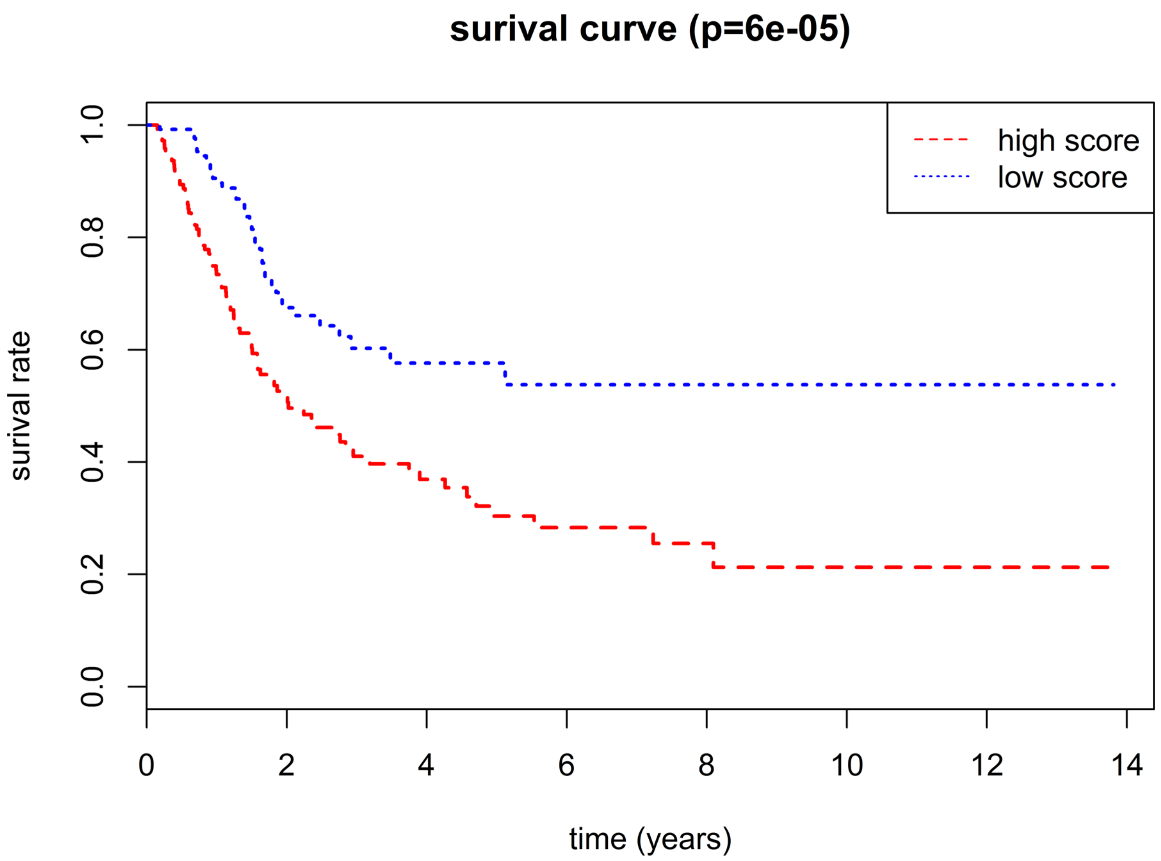

Supplementary Figure 4: Kaplan–Meier curve for the three-miRNA signature and the survival time of patients with BLCA in male group.

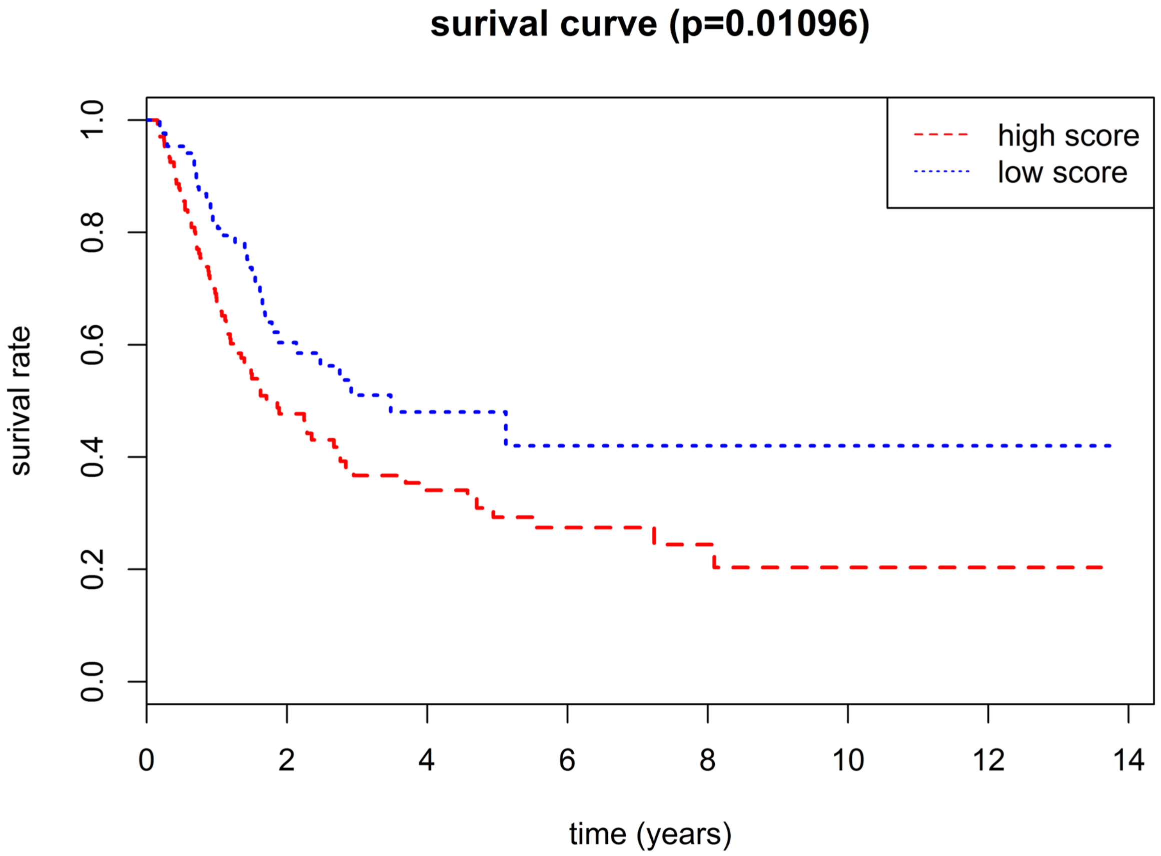

Supplementary Figure 5: Kaplan–Meier curve for the three-miRNA signature and the survival time of patients with BLCA in the group of who are more than 65 years old.

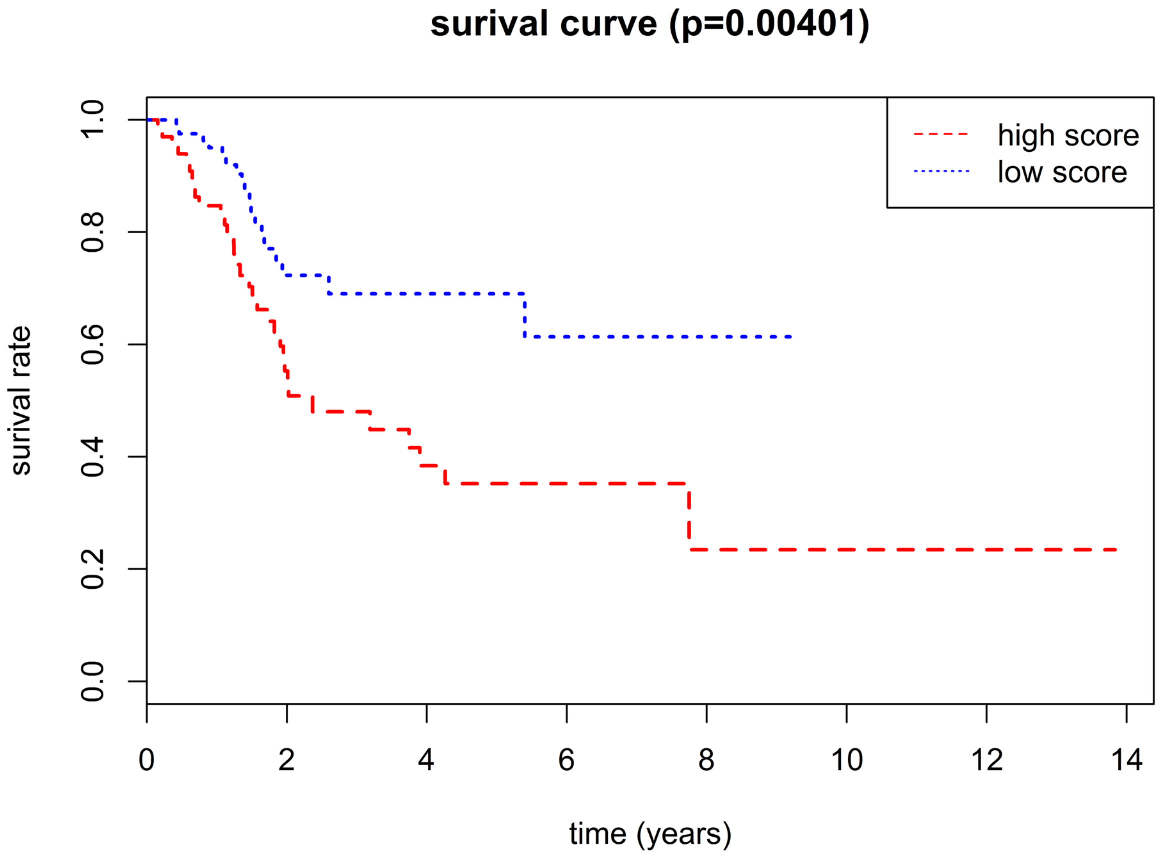

Supplementary Figure 6: Kaplan–Meier curve for the three-miRNA signature and the survival time of patients with BLCA in the group of who are less than 65 years old.

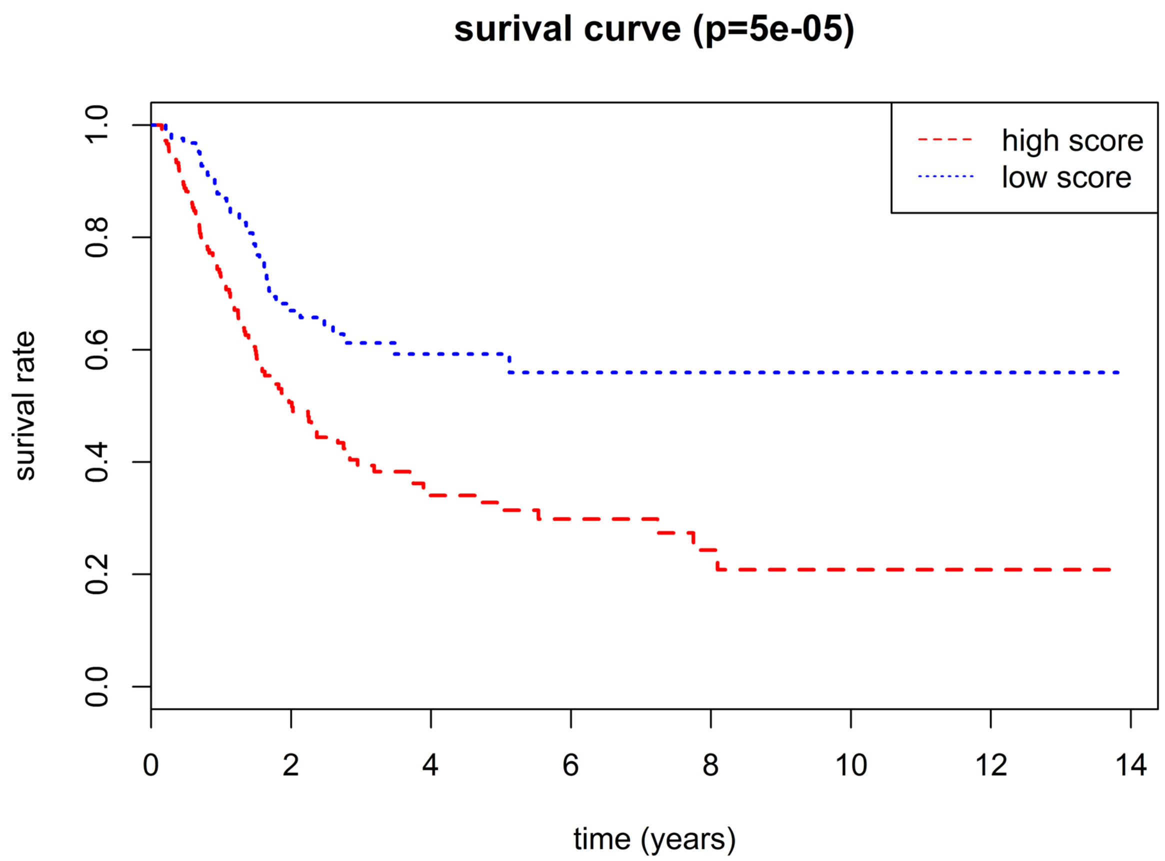

Supplementary Figure 7: Kaplan–Meier curve for the three-miRNA signature and the survival time of patients with BLCA in Caucasians group.

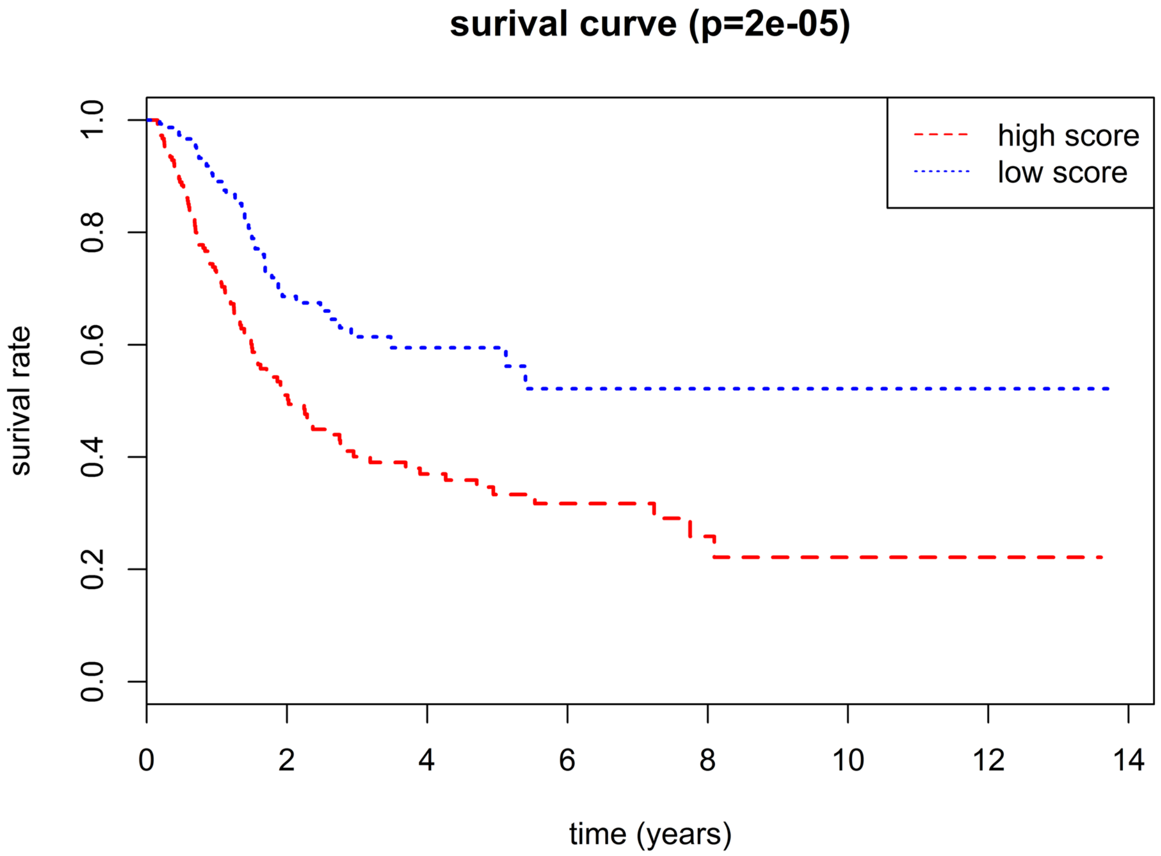

Supplementary Figure 8: Kaplan–Meier curve for the three-miRNA signature and the survival time of patients with BLCA in the group without radiation therapy.

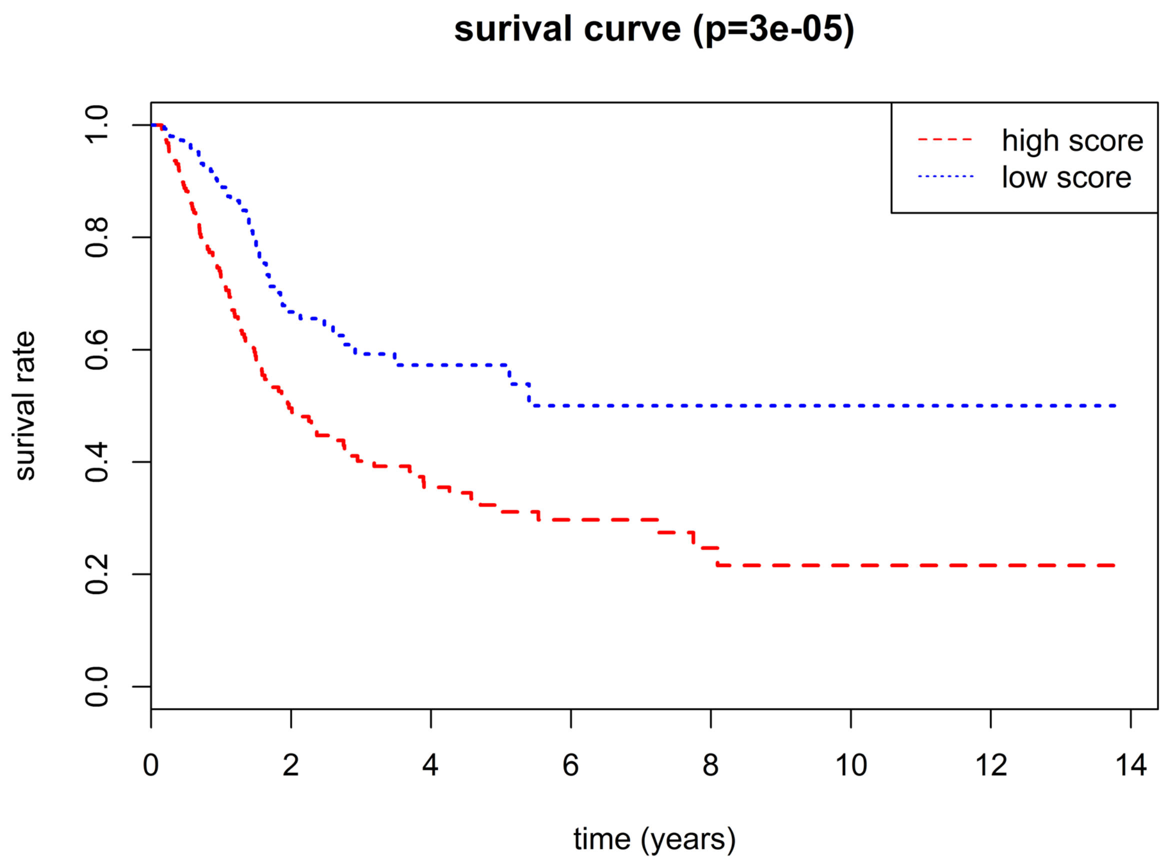

Supplementary Figure 9: Kaplan–Meier curve for the three-miRNA signature and the survival time of patients with BLCA in the group of T stage.

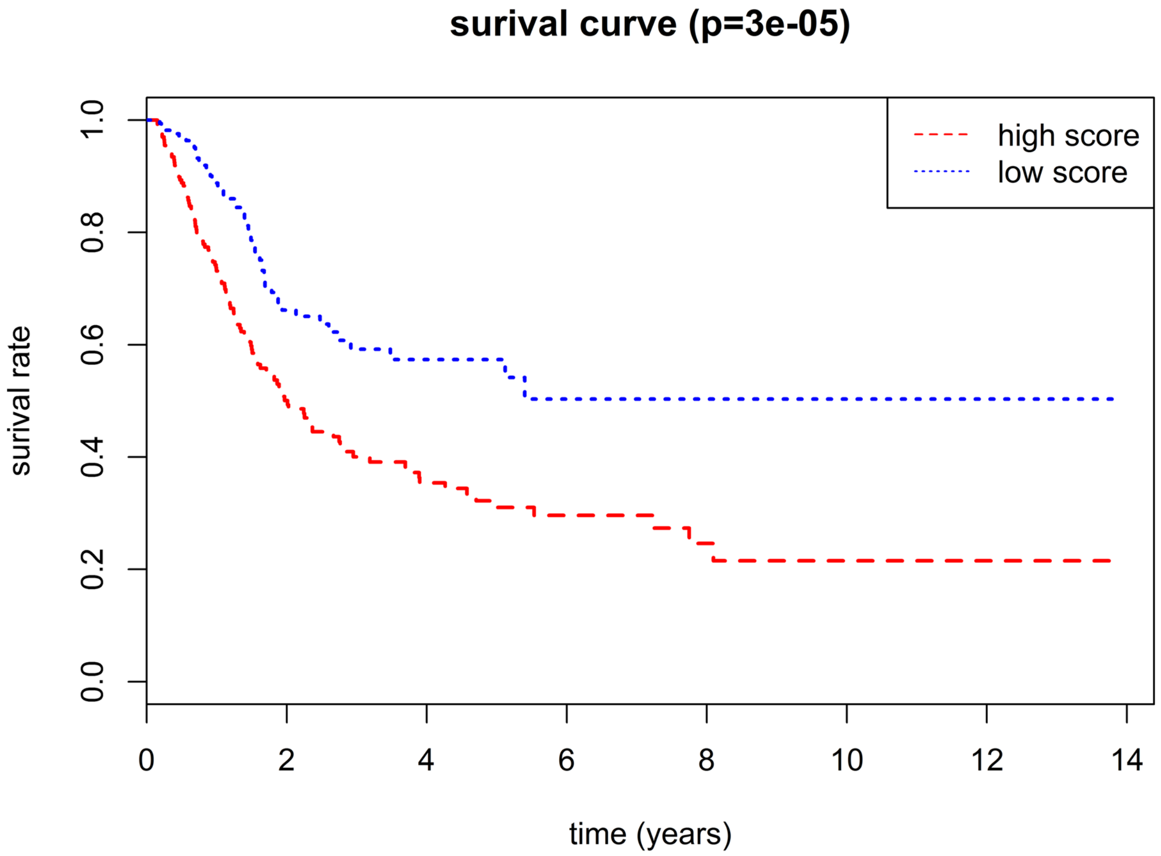

Supplementary Figure 10: Kaplan–Meier curve for the three-miRNA signature and the survival time of patients with BLCA in the group of N stage.

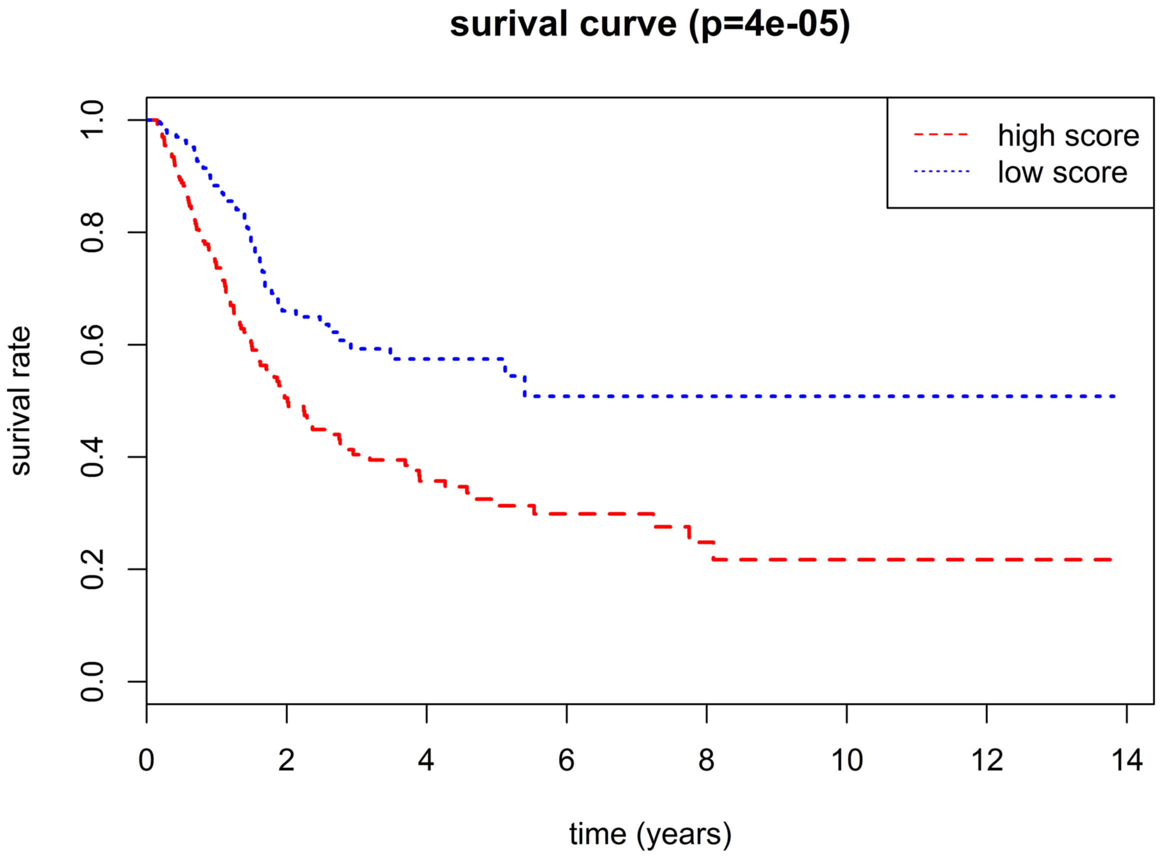

Supplementary Figure 11: Kaplan–Meier curve for the three-miRNA signature and the survival time of patients with BLCA in the group of M stage.

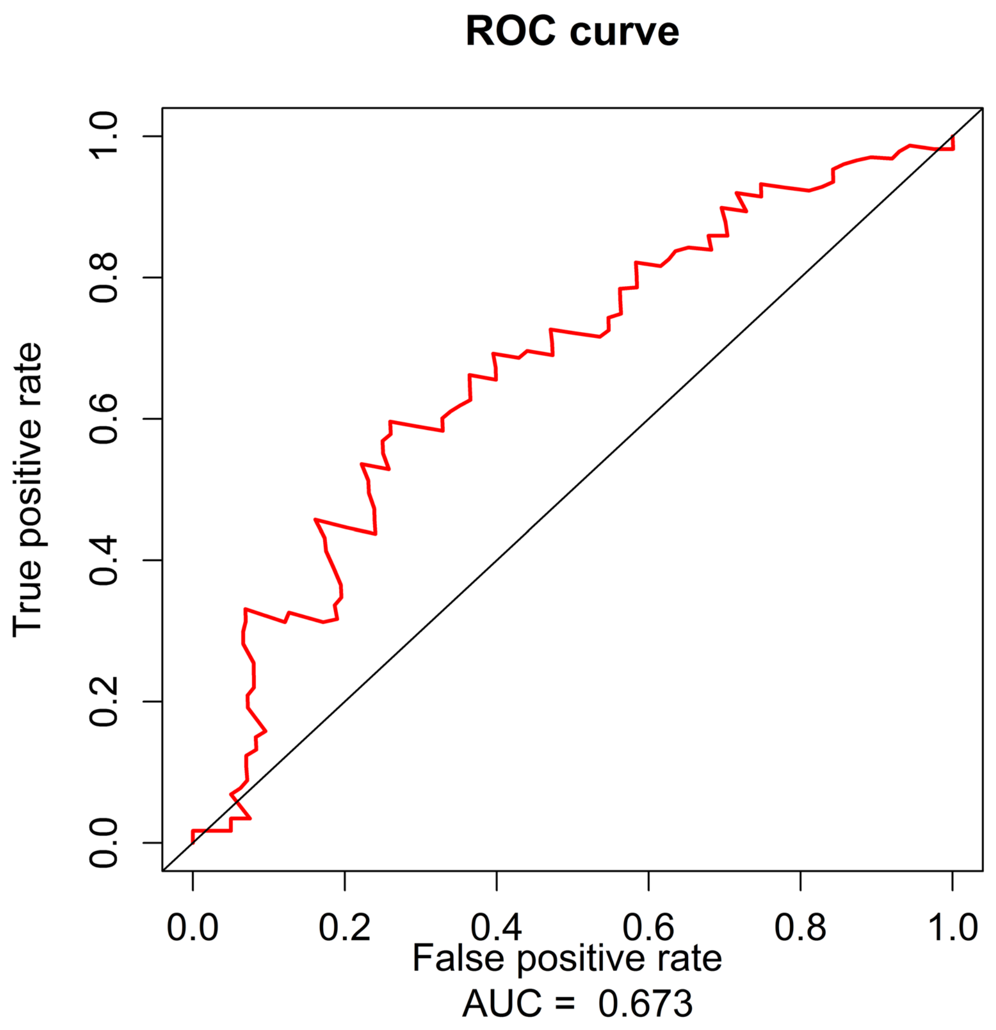

Supplementary Figure 12: ROC curves for the three-miRNA signature in predicting 5-year survival rate in the famale group of BLCA patients.

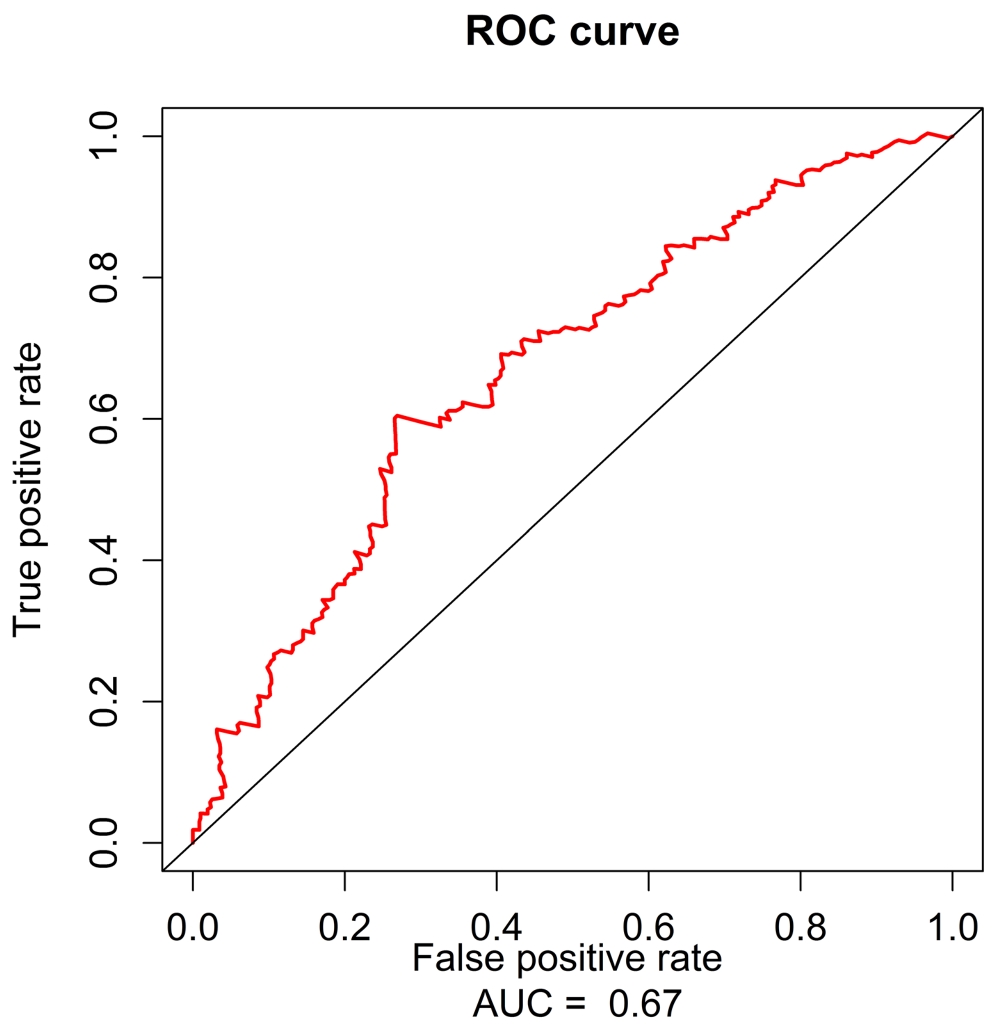

Supplementary Figure 13: ROC curves for the three-miRNA signature in predicting 5-year survival rate in the male group of BLCA patients.

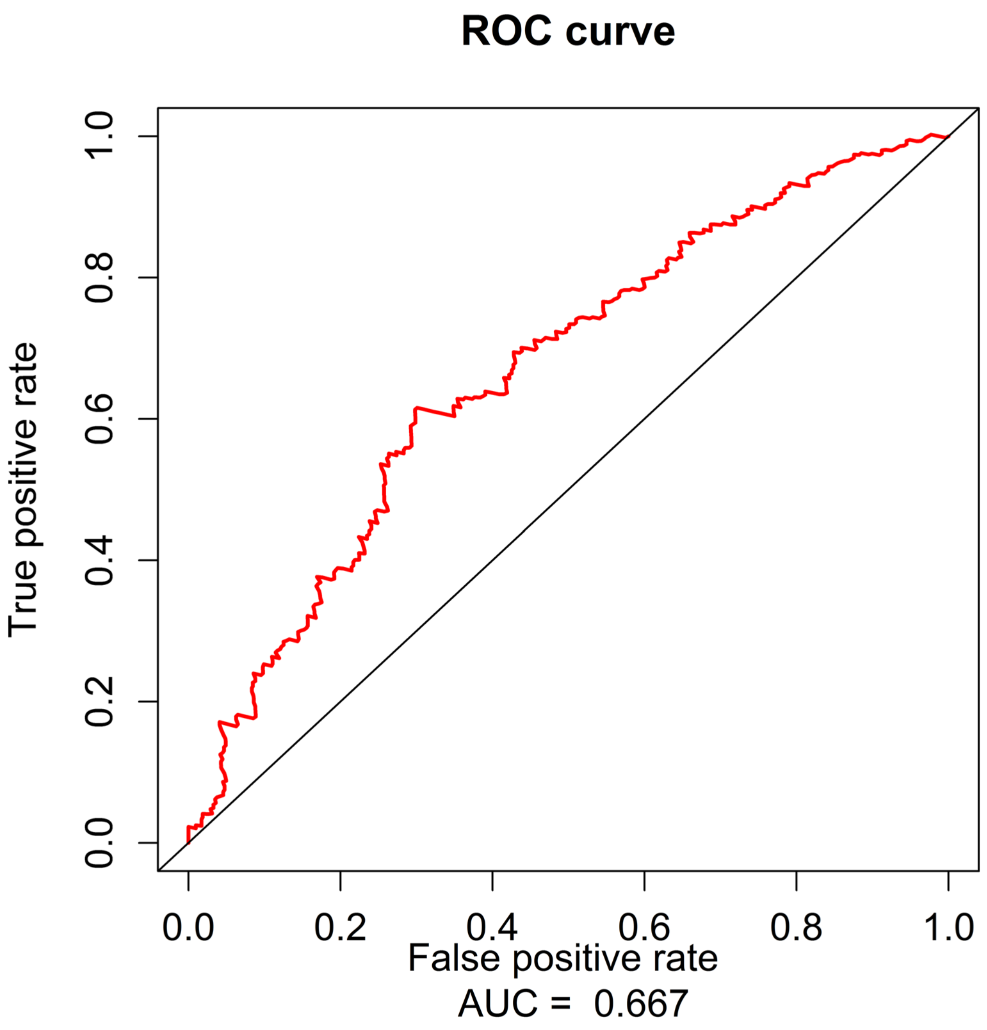

Supplementary Figure 14: ROC curves for the three-miRNA signature in predicting 5-year survival rate in the M stage group of BLCA patients.

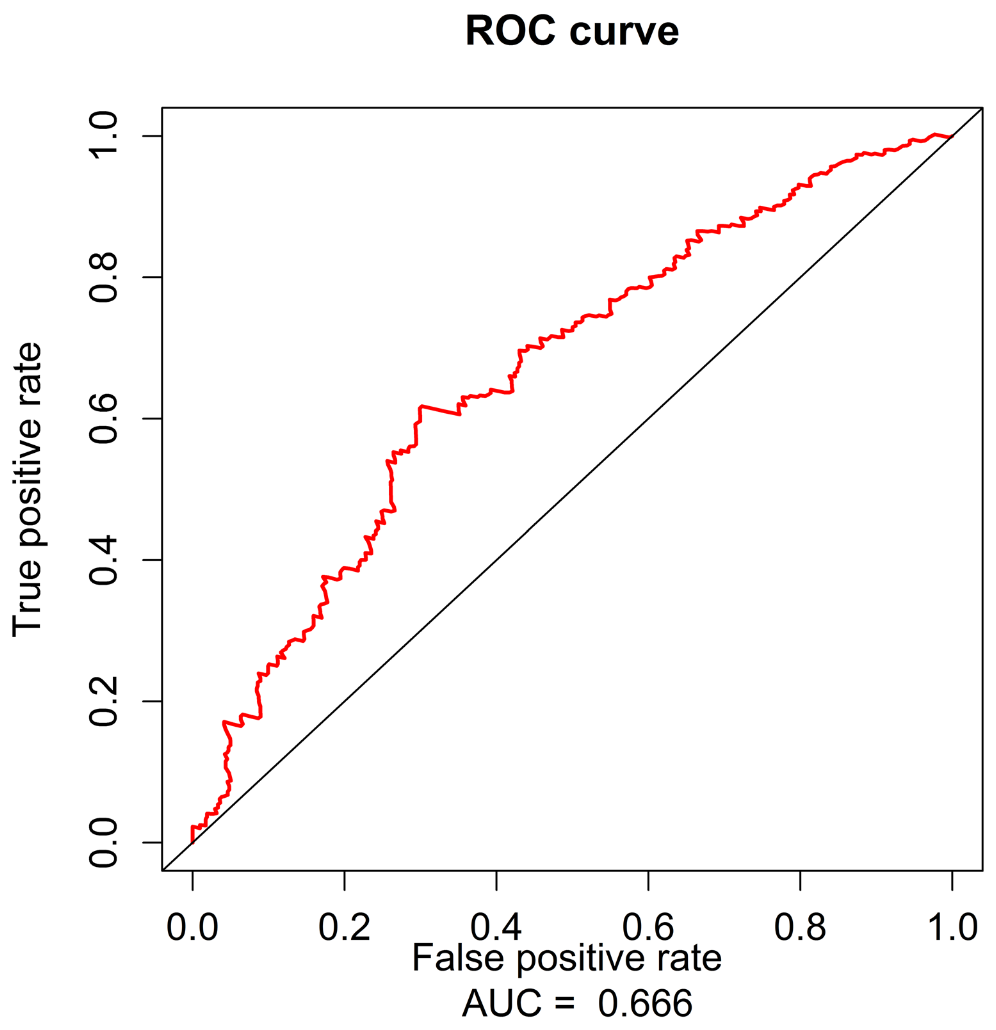

Supplementary Figure 15: ROC curves for the three-miRNA signature in predicting 5-year survival rate in the N stage group of BLCA patients.

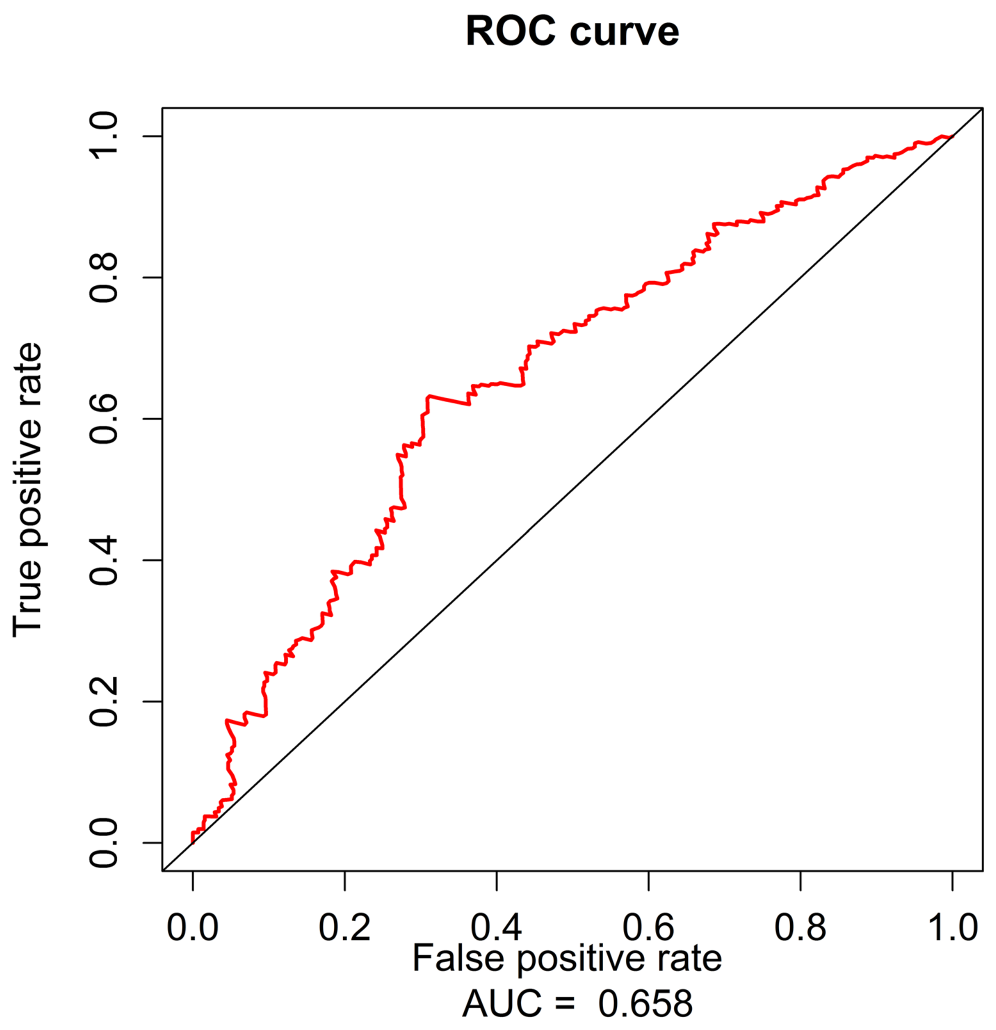

Supplementary Figure 16: ROC curves for the three-miRNA signature in predicting 5-year survival rate in the T stage group of BLCA patients.

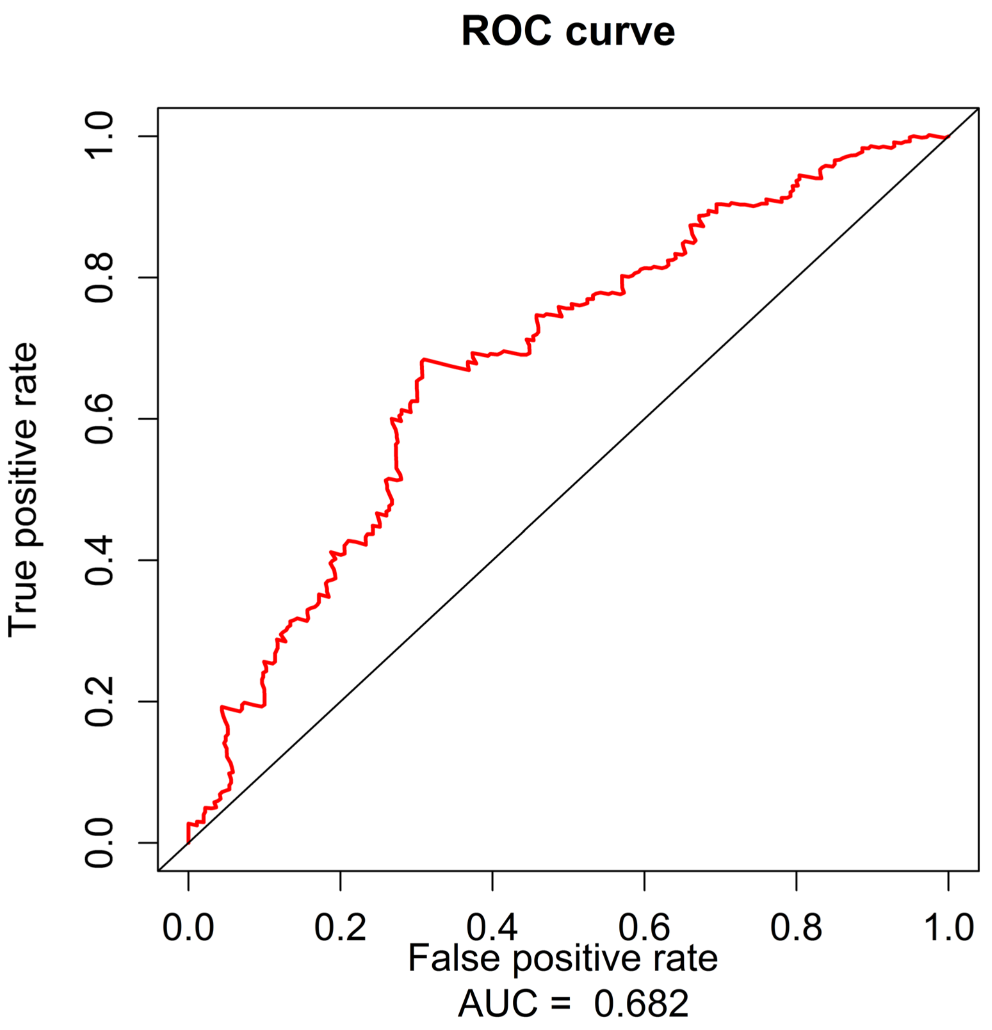

Supplementary Figure 17: ROC curves for the three-miRNA signature in predicting 5-year survival rate in the Caucasians group of BLCA patients.

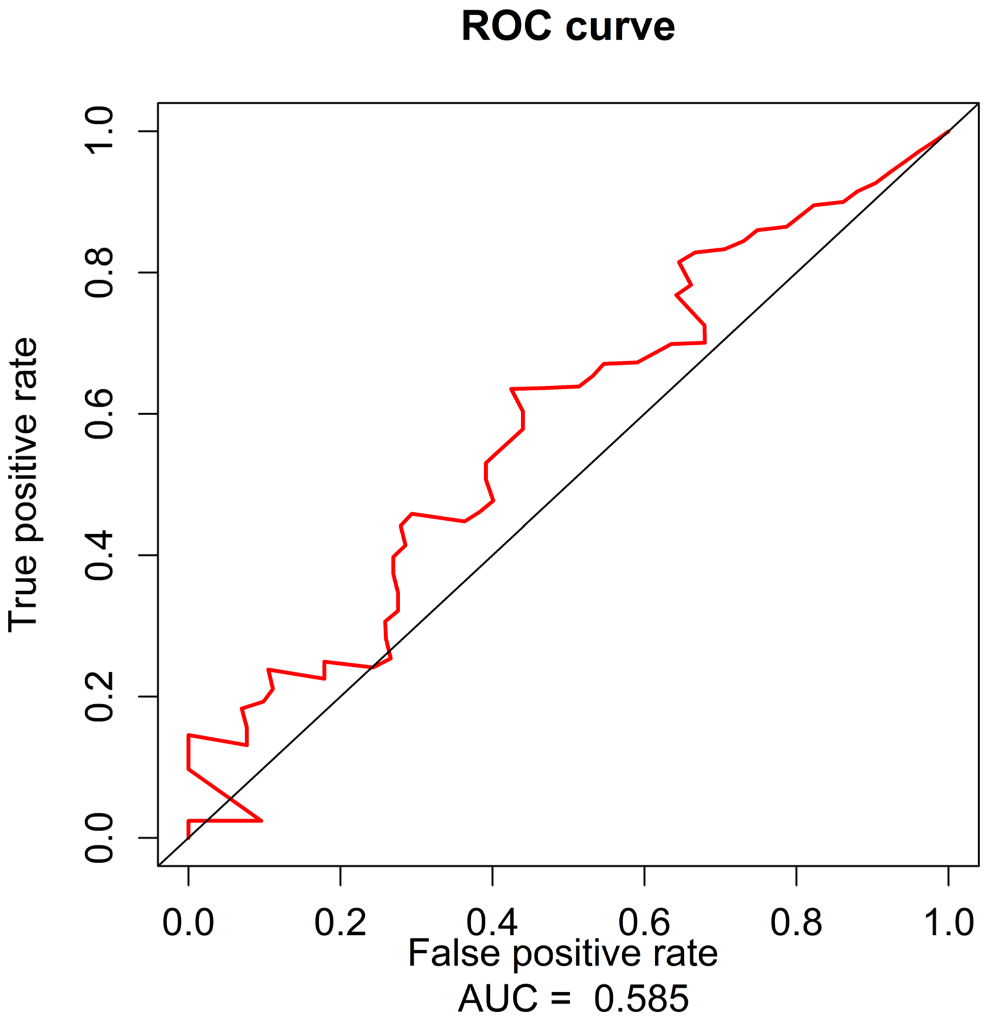

Supplementary Figure 18: ROC curves for the three-miRNA signature in predicting 5-year survival rate in the non-Caucasians group of BLCA patients.

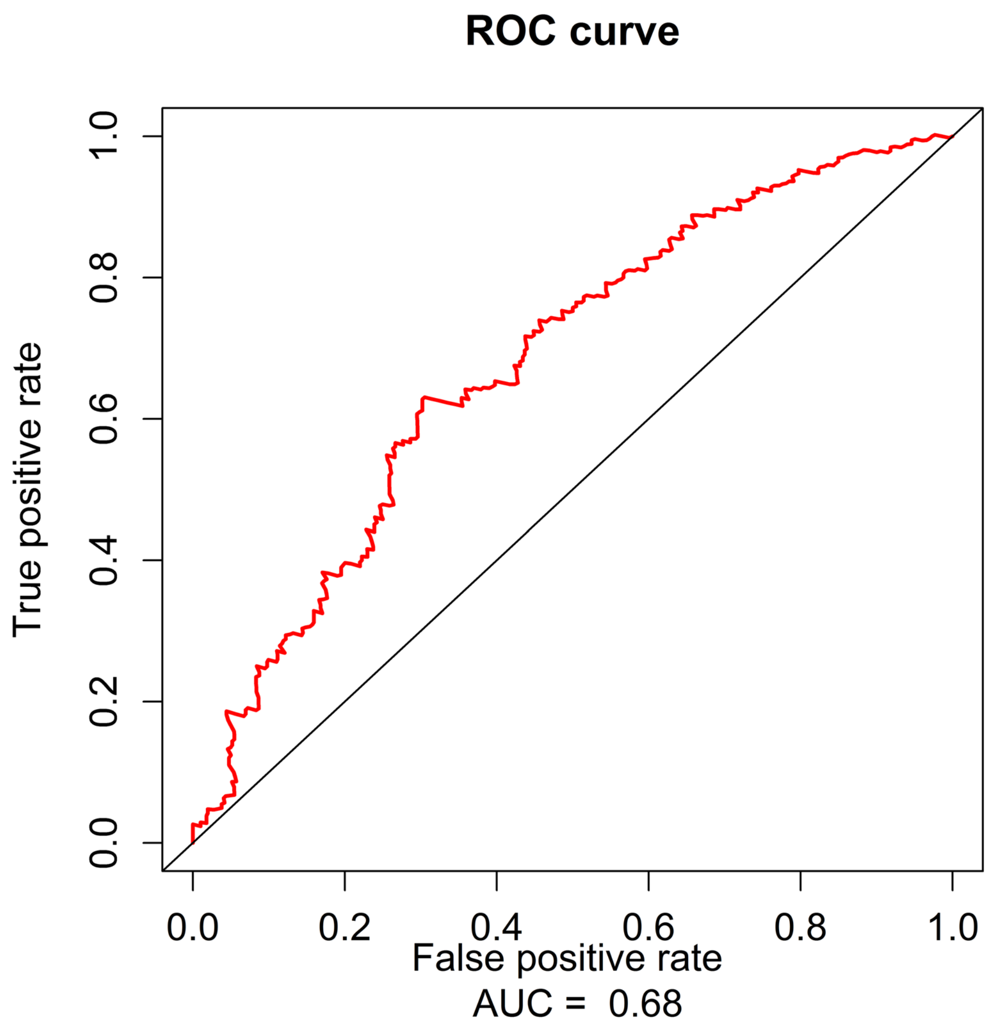

Supplementary Figure 19: ROC curves for the three-miRNA signature in predicting 5-year survival rate in the group without radiation therapy of BLCA patients.

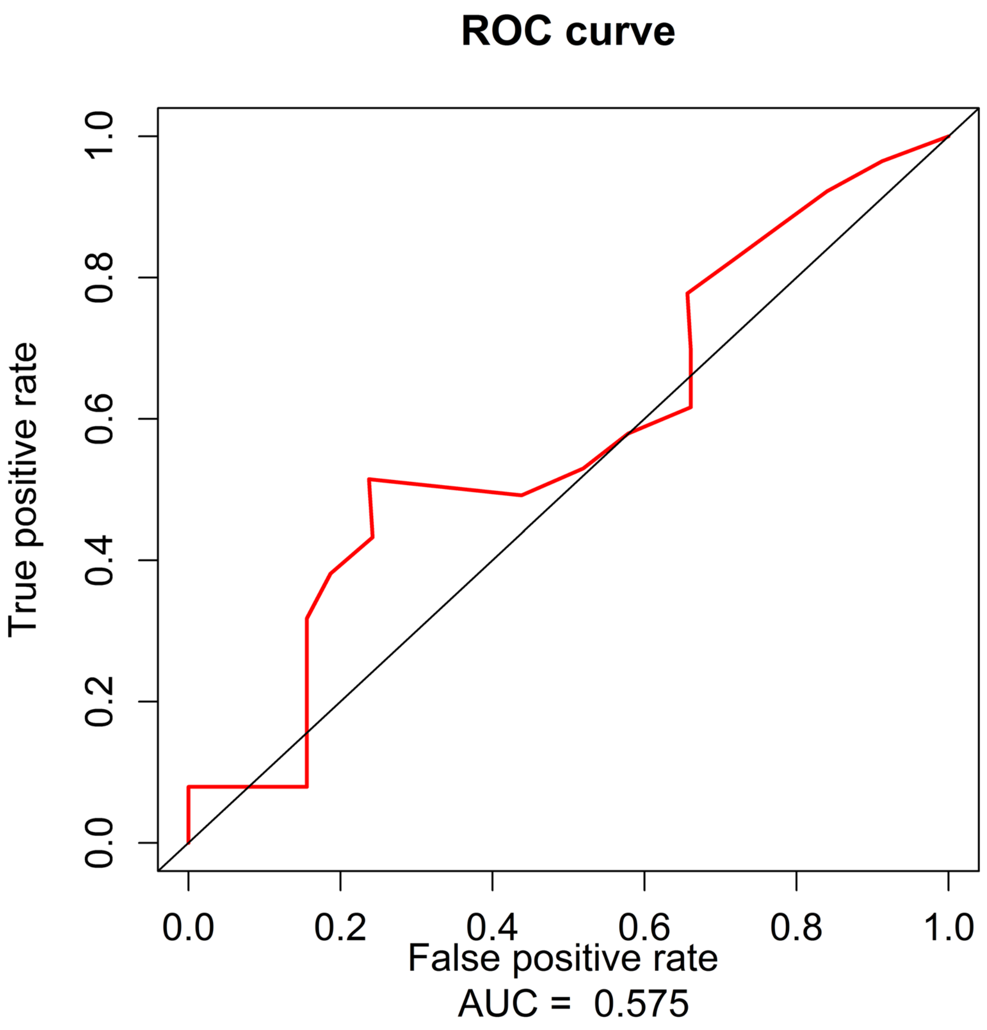

Supplementary Figure 20: ROC curves for the three-miRNA signature in predicting 5-year survival rate in the group with radiation therapy of BLCA patients.

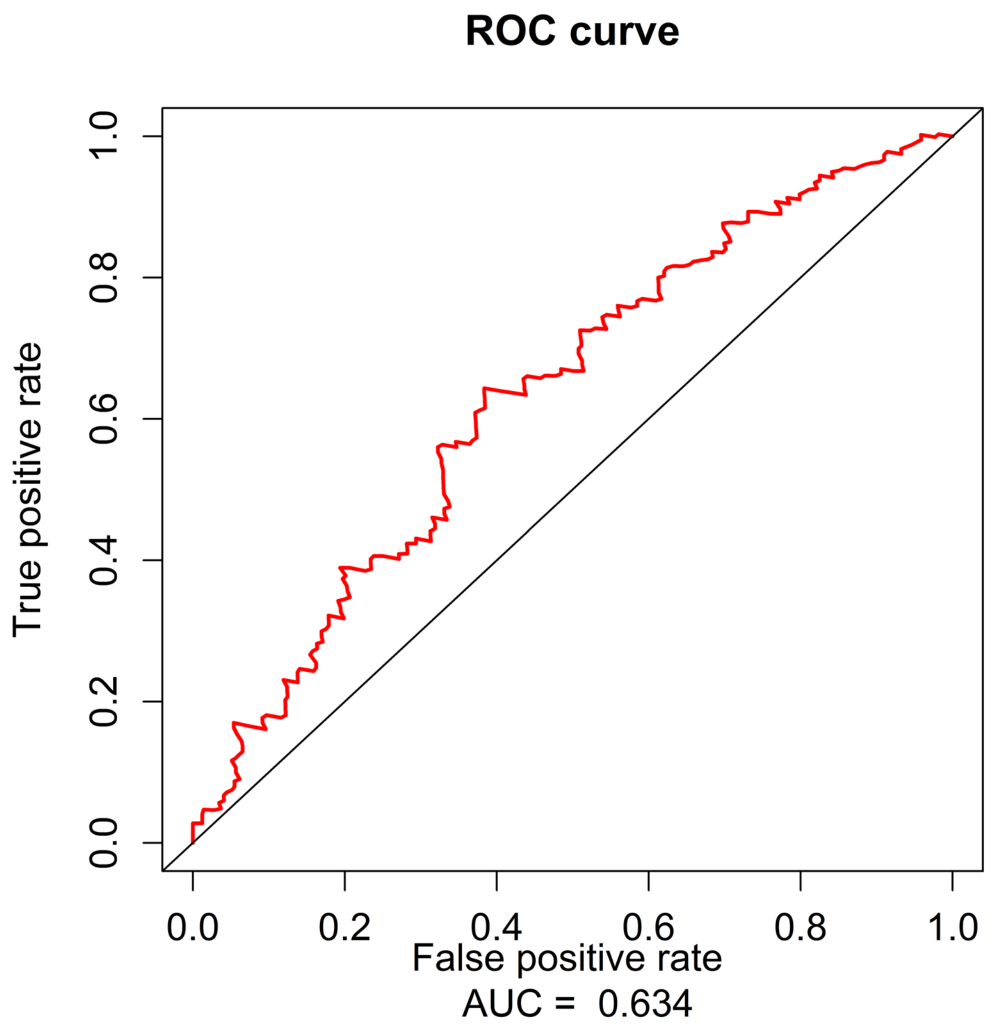

Supplementary Figure 21: ROC curves for the three-miRNA signature in predicting 5-year survival rate in the group of who are more than 65 years old of BLCA patients.

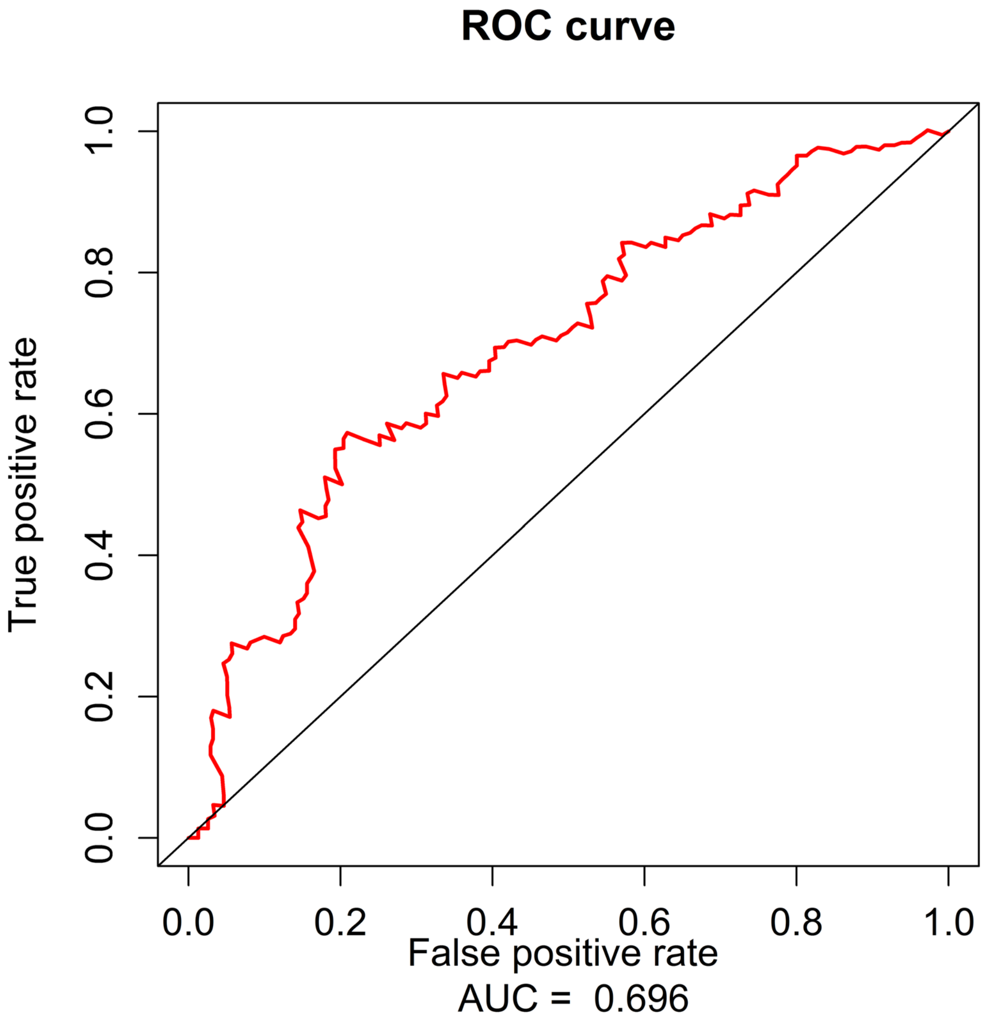

Supplementary Figure 22: ROC curves for the three-miRNA signature in predicting 5-year survival rate in the group of who are less than 65 years old of BLCA patients.

Supplementary Table 1: 295 miRNAs were overexpressed in BLCA patients

See Supplementary File 1

Supplementary Table 2: 97 miRNAs were downexpressed in BLCA patients

See Supplementary File 2

Supplementary Table 3: Target of three miRNA signature in BLCA patients

See Supplementary File 3

Supplementary Table 4: GO term by target genes of three miRNA signature in BLCA patients

See Supplementary File 4

Supplementary Table 5: KEGG pathway of three miRNA signature

See Supplementary File 5
